# Primary Meningeal Melanocytic Tumors of the Central Nervous System: A Review from the Ultra-Rare Brain Tumors Task Force of the European Network for Rare Cancers (EURACAN)

**DOI:** 10.3390/cancers16142508

**Published:** 2024-07-10

**Authors:** Alessia Pellerino, Robert M. Verdijk, Lucia Nichelli, Nicolaus H. Andratschke, Ahmed Idbaih, Roland Goldbrunner

**Affiliations:** 1 Division of Neuro-Oncology, Department of Neuroscience “Rita Levi Montalcini”, University and City of Health and Science Hospital, 10126 Torino, Italy; 2 Department of Pathology, Section Ophthalmic Pathology, Erasmus MC University Medical Center, 3015 Rotterdam, The Netherlands; r.verdijk@erasmusmc.nl; 3 Department of Pathology, Leiden University Medical Center, 2333 Leiden, The Netherlands; 4 Department of Neuroradiology, Assistance Publique-Hôpitaux de Paris, Sorbonne Université, Groupe Hospitalier Pitié-Salpêtrière-Charles Foix, 75013 Paris, France; lucia.nichelli@aphp.fr; 5 Department of Radiation Oncology, University of Zurich, University Hospital Zurich, 8091 Zurich, Switzerland; nicolaus.andratschke@usz.ch; 6 CNRS, Inserm, DMU Neurosciences, Service de Neuro-Oncologie-Institut de Neurologie, Sorbonne Université, Hôpitaux Universitaires La Pitié Salpêtrière-Charles Foix, F-75013 Paris, France; ahmed.idbaih@aphp.fr; 7 Center for Neurosurgery, Department of General Neurosurgery, University of Cologne, 50923 Cologne, Germany; roland.goldbrunner@uk-koeln.de

**Keywords:** EURACAN, diffuse leptomeningeal melanocytosis or melanomatosis, meningeal melanocytoma, meningeal melanoma, melanocytic tumor

## Abstract

**Simple Summary:**

The European Network for Rare Cancers (EURACAN) Task Force on Ultra-Rare Brain Tumors (domain 10, subdomain 10) has reviewed the literature extensively regarding primary meningeal melanocytic tumors, which are ultra-rare entities that affect primarily children and young adults and represent an unmet need in neuro-oncology. The review covers the main histological and molecular factors, radiological findings, clinical features, and therapeutic challenges. Further efforts are needed to collect more knowledge on these rare brain tumors to improve the diagnostic tools and provide novel treatment strategies.

**Abstract:**

Background: Primary meningeal melanocytic tumors are ultra-rare entities with distinct histological and molecular features compared with other melanocytic or pigmented lesions, such as brain and leptomeningeal metastases from metastatic melanoma. Methods: The European Network for Rare Cancers (EURACAN) Task Force on Ultra-Rare Brain Tumors (domain 10, subdomain 10) performed a literature review from January 1985 to December 2023 regarding the epidemiologic and clinical characteristics, histological and molecular features, radiological findings, and efficacy of local treatments (surgery and radiotherapy) and systemic treatments for these entities. Results: Molecular analysis can detect specific mutations, including GNAQ, GNA11, SF3B1, EIF1AX, BAP1, that are typically found in circumscribed primary meningeal melanocytic tumors and not in other melanocytic lesions, whereas NRAS and BRAF mutations are typical for diffuse primary meningeal melanocytic tumors. The neuroimaging of the whole neuroaxis suggests a melanocytic nature of a lesion, depicts its circumscribed or diffuse nature, but cannot predict the tumor’s aggressiveness. Gross-total resection is the first choice in the case of circumscribed meningeal melanocytoma and melanoma; conversely, meningeal biopsy may be reserved for patients with diffuse and multinodular leptomeningeal spread to achieve a definitive diagnosis. High-dose radiotherapy is rarely indicated in diffuse melanocytic tumors except as palliative treatment to alleviate symptoms. Last, a definitive advantage of a specific systemic treatment could not be concluded, as most of the data available derive from case reports or small cohorts. Conclusions: As primary meningeal melanocytic tumors are extremely rare, the correlations between the clinical characteristics, molecular profile, radiological findings at diagnosis and progression are weak, and poor evidence on the best therapeutic approach is available. There is a need to develop shared platforms and registries to capture more knowledge regarding these ultra-rare entities.

## 1. Introduction

Neuro-oncologists are often involved in the management of ultra-rare brain tumors, where guidelines, recommendations or strong evidence on how to diagnose or treat these diseases are significantly poor. Primary meningeal melanocytic tumors of the central nervous system (CNS) are the paradigm of these conditions and include a spectrum of extremely rare tumors that arise from melanocytes originating from the neural crest that invade the leptomeninges or the choroid plexus during embryonic development. Primary meningeal melanocytic tumors are considered extremely rare since they are not captured by prospective tumor registries; therefore, it is difficult to estimate the exact population-based incidence. In a retrospective cohort of 116 patients with primary or metastatic melanotic lesions of the CNS collected by the Yale Cancer Center between 2001 and 2019, only four patients who met the histological criteria of primary meningeal melanocytic tumors were identified, reflecting a relative incidence of 3.4% [1]. The World Health Organization (WHO) 2021 Classification of CNS Tumors identified a presentation either as a circumscribed, solitary, and bulky leptomeningeal lesion or a diffuse/multifocal leptomeningeal dissemination [2]. In general, melanoblasts can give origin to four different melanocytic tumors. A differentiated, circumscribed melanocytic tumor (benign) is known as meningeal melanocytoma, while the malignant counterpart is represented by the meningeal melanoma. Meningeal melanocytomas and meningeal melanomas account for 0.06–0.1% of the total meningeal tumors. Meningeal melanocytomas have an estimated incidence of 1/10,000,000 person-year, while an annual incidence of 0.005 cases per 100,000 population has been reported for meningeal melanomas [3]. Of note, meningeal melanomas differ from brain metastases from melanomas since there is never evidence of systemic localizations of melanomas (e.g., cutaneous, uveal or gastrointestinal primary localizations) and have a distinct molecular pattern (see Section 3). The spread of melanoblasts along meninges may lead to multiple linear or nodular lesions along the cranial and/or spinal leptomeninges, known as diffuse meningeal melanocytic tumors. Based on the histological features, a benign or a malignant phenotype may be recognized, known as primary diffuse leptomeningeal melanocytosis or melanomatosis, respectively [2,4]. Overall, diffuse meningeal melanocytic tumors are considered ultra-rare entities since the exact population-based incidence is unknown, and the current knowledge of these peculiar entities is based on few case reports. Thus, there is a lack of large cohorts that describe the correlations between histology, molecular features, radiological findings, and clinical course. Traditionally, primary meningeal melanocytic tumors do not have specific clinical peculiarities. MRI can suggest the melanocytic nature of a lesion but does not distinguish between a primary or a secondary melanocytic tumor. Therefore, remarkable diagnostic and therapeutic challenges exist for clinicians, and a deeper understanding of the nature and evolution of these entities is urgently needed.

Based on this scenario, the European Network for Rare Cancers (EURACAN) Task Force on Ultra-Rare Brain Tumors (domain 10, subdomain 10) has conducted an extensive review of the epidemiologic and clinical characteristics, histological and molecular features, radiological findings, and efficacy of local treatments, including surgery and radiotherapy, and systemic therapies.

A literature search was conducted for studies published from January 1985 to December 2023 in the PubMed, Scopus, Web of Science Core Collection, and Embase database using the following keywords: “melanocytic tumors” and/or “primary melanocytic tumors” and/or “meningeal melanocytic tumors” and/or “meningeal melanocytoma” and/or “meningeal melanoma” and/or “diffuse meningeal melanocytosis” and/or “diffuse meningeal melanomatosis”. Published conference abstracts were also included. The authors have included in the literature search patients of all ages without evidence of extracranial metastasizing primary tumors (e.g., metastatic melanoma). Cases with a diffuse meningeal melanocytic tumor with ≥1 nodular lesion with a leptomeningeal base implant were also included. All abstracts were screened by A.P., in addition to one other reviewer according to individual specialty (A.I. for “clinical features” and “medical treatments”; R.M.V. for “pathology and molecular markers”; L.N. for “neuroimaging”; R.G. for “surgery”; N.H.A. for “radiotherapy”). Conflicts were resolved by internal discussion between reviewers; a consensus was always achieved, and a third reviewer was never needed to solve conflicts.

## 2. Clinical Features

Primary meningeal melanocytic tumors can be part of the clinical manifestations of neurocutaneous melanosis, a sporadic and rare congenital neurological disorder, characterized by abnormal aggregations of nevomelanocytes within the CNS (leptomeningeal melanocytosis) associated with large or giant congenital melanocytic nevi. The giant congenital melanocytic nevus is a pigmented skin lesion >20 cm of diameter (40 cm for “giant”) in adult patients, composed of aggregated melanocytes in a delimited area of the body with overlying hypertrichosis, with an increased risk of malignant transformation. Moreover, giant congenital nevi present a distinctive unique distribution that provides a “garment” appearance as an expression of the developmental disorder of the melanocyte precursors from the neural crest [5]. Neurocutaneous melanosis has an estimated prevalence of 1/50,000–1/200,000 and an incidence of 0.5–2 new cases per 100,000 person-year. Typically, neurocutaneous melanosis patients without leptomeningeal involvement are asymptomatic and show a normal life expectancy. Conversely, symptomatic neurocutaneous melanosis have a poor prognosis due to the development of neurological complications, with a significant association with Dandy–Walker or Chiari malformations, hydrocephalus, and primary meningeal melanocytic tumor-related symptoms [6]. Approximately 10–15% of patients with neurocutaneous melanosis syndrome develop meningeal melanocytomas [7] and another 23% present a radiological CNS involvement without any symptoms [8]. Primary meningeal melanocytic tumors can also occur in patients with the BAP1 tumor predisposition syndrome (germline BAP1 mutation) that confers a higher risk of meningeal, uveal, and cutaneous melanomas, as well as other tumor types, including mesotheliomas and clear cell renal cancers [9].

Meningeal melanocytomas can occur at any age with a prevalence in the fourth to fifth decades and a median age ranging from 45.6 years to 53.7 years [10,11], while the median age at diagnosis of meningeal melanomas is 48 years in adult populations and 3.0 years for children [12,13]. Circumscribed lesions (meningeal melanocytomas and melanomas) are often located in the posterior cranial base, in proximity to the foramen magnum, or in the trigeminal cave, due to a higher, physiological melanocytic density in these locations [14]. Meningeal melanocytomas may be associated with nevus of Ota, or ocular dermal melanosis, which is a benign melanosis that involves the distribution of the trigeminal nerve, mainly the ophthalmic and maxillary divisions, with associated hyperpigmentation of the eye and adnexa [15]. The intraventricular or intramedullary localization of meningeal melanocytoma or melanoma is particularly rare [16,17], while a CNS-isolated intraparenchymal presentation should firstly suggest a metastatic melanoma. Meningeal melanomas may rarely spread extracranially with bone, liver, and lung metastases [18,19].

Primary diffuse leptomeningeal melanocytosis is often associated with large/giant congenital melanocytic nevi and may have an aggressive clinical course. Neurological symptoms depend on the site of presentation, and progress rapidly in the case of malignant transformation (primary diffuse leptomeningeal melanomatosis), with seizures, increased intracranial pressure and hydrocephalus; focal symptoms including aphasia, motor or sensory deficits, scotoma/blurred vision/impaired visual field, cerebellar signs, gait disturbance and ataxia. Similarly, spinal symptoms reflect the level of spine compression and leptomeningeal involvement with para- or tetraparesis, sensory levels, radicular and/or back pain, urinary or bowel disturbances [3,20]. Primary diffuse leptomeningeal melanocytosis patients can be asymptomatic for a variable period of time, but when neurological symptoms have developed, the prognosis is remarkably poor [18]. Primary diffuse leptomeningeal melanomatosis is a devastating disease with a rapid evolution and a dismal prognosis [21]. The increased intracranial pressure, hydrocephalus, headache and meningeal and radicular symptoms are the main neurological complications, and the ventriculoperitoneal shunting provides a palliative effect, but with a high risk of peritoneal melanomatosis [22].

## 3. Pathology and Molecular Markers

**Figure 1:**
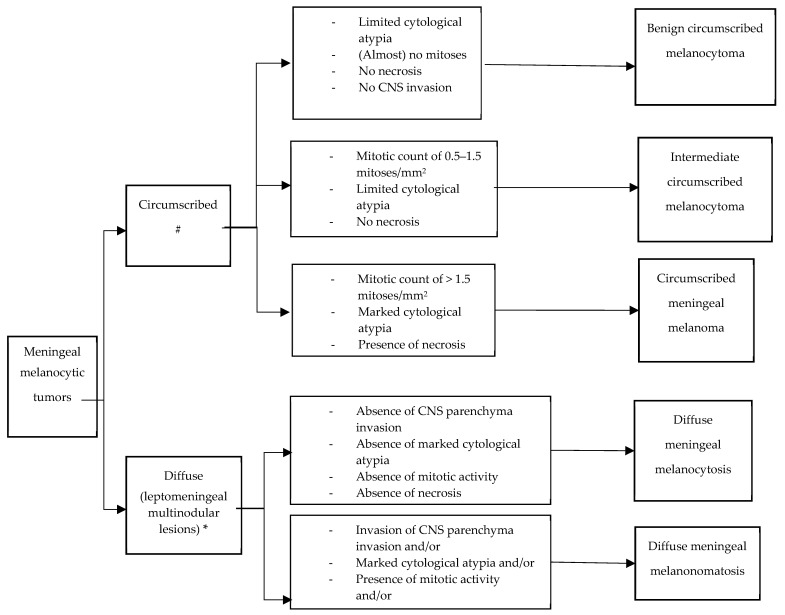
Flowchart and recommendations for pathological diagnosis according to WHO 2021. Desirable: * NRAS or rarely BRAF mutation. # GNAQ, GNA11, PLCB4 or CYSLTR2 mutation. Additional markers BAP1, SF3B1, EIF1AX mutations, monosomy of chromosome 3 and complex CNV indicate aggressive behavior.

Primary meningeal melanocytic tumors, either circumscribed or diffuse, show a spectrum of histopathological features, ranging from blue nevus-like, well-differentiated neoplasms to melanomas. A summary of the essential diagnostic criteria according to the WHO Classification of Tumors of the CNS 5th edition, for the tumors covered in this guideline is presented in the Figure 1.

### 3.1. Circumscribed Meningeal Melanocytic Neoplasms

Meningeal melanocytoma is a circumscribed melanocytic neoplasm with a bland histological appearance. The term “circumscribed” refers to the absence of any infiltration of structures beyond the pia mater or Virchow Robin spaces and the absence of invasion of glial tissues. Tumors with increased mitotic activity or invasion of CNS parenchyma have been defined as melanocytoma of intermediate grade [23]. Well-differentiated melanocytomas may show variable cell density but are usually composed of densely packed, slightly spindled or oval tumor cells containing variable (often abundant) melanin. Heavily pigmented tumor cells and melanophages are especially seen at the periphery of nests. Seldom have amelanotic melanocytomas been described [24]. The nuclei are oval or bean-shaped, occasionally showing grooves, with small eosinophilic nucleoli (spindle type A). Cytological atypia, necrosis, and mitoses are typically absent (on average <0.5 mitoses/mm^2^, equating to <1 mitosis/10 HPF of 0.5 mm in diameter and 0.2 mm^2^ in area, Table 1). Meningeal melanocytomas generally do not show invasion of CNS parenchyma [14]. According to the WHO definition, meningeal melanocytic tumors with the histology of melanocytoma but showing CNS invasion or increased mitotic activity (0.5–1.5 mitoses/mm^2^, equating to 1–3 mitoses/10 HPF of 0.5 mm in diameter and 0.2 mm^2^ in area) have been defined as intermediate-grade melanocytic neoplasms [14] (Figure 1).

Meningeal melanoma is a malignant circumscribed meningeal melanocytic neoplasm that arises from leptomeningeal melanocytes with aggressive growth properties [23]. Meningeal melanomas are composed of pleomorphic spindled (spindle type B) and/or epithelioid cells with variable cytoplasmic melanin [14] and polymorphic nuclei that have large irregular eosinophilic nucleoli. Numerous (>1.5/mm^2^) mitotic figures can be seen. They often demonstrate unequivocal invasion of the CNS parenchyma and/or necrosis. Meningeal melanoma may spread through the subarachnoid space. The melanocytes of circumscribed meningeal melanocytic neoplasms express common melanocytic markers by immunohistochemistry. Hemizygous mutations of BAP1, combined with chromosome 3 monosomy, can be revealed by absent BAP1 protein expression.

Molecular pathology is comparable to uveal melanoma [42], with mutually exclusive activating hotspot mutations in GNAQ, GNA11, PLCB4, and CYSLTR2 that are considered the first step in the oncogenesis of circumscribed meningeal melanocytic tumors [18,43]. GNAQ and GNA11 mutations are most frequent, observed in about 60–70% of cases [10,11]. Meningeal melanocytomas and melanomas do not usually harbor HRAS, KRAS, BRAF, or KIT mutations [10,44,45,46]. TERT promoter mutations are also usually absent [47]. Likewise, in uveal melanomas, a mutation in EIF1AX, SF3B1, or BAP1 is considered the next step in the oncogenic process [10,11,42,48,49]. The possible adverse prognostic relevance of these mutations, well described in uveal melanoma [48], requires further validation in meningeal melanocytoma of intermediate grade and melanoma [49]. The chromosomal alterations and methylation pattern identified in circumscribed meningeal melanocytic neoplasms, uveal melanomas and blue nevus–like melanoma show common DNA-methylation and copy-number alterations [11,50].

### 3.2. Diffuse Meningeal Melanocytic Neoplasms

Meningeal melanocytosis is a diffuse or multifocal neoplasm of cytologically bland melanocytic cells of variable shape and pigmentation that remain within the subarachnoid and Virchow–Robin spaces [23]. Nuclei are bland with inconspicuous nucleoli. Histologically bland lesions that show unequivocal invasion of the CNS parenchyma should be considered as meningeal melanomatosis [51].

Meningeal melanomatosis is a diffuse or multifocal proliferation of melanoma cells that often shows CNS invasion [23]. Nuclei generally show marked atypia with large eosinophilic nucleoli. Mitoses and/or necrosis also warrant a diagnosis of meningeal melanomatosis [52]. The melanocytes of meningeal melanocytosis and melanomatosis express common melanocytic markers by immunohistochemistry. However, it should be remembered that SOX10 and S100 will not discriminate melanocytes from astrocytes.

Molecular pathology shows that both cutaneous and diffuse meningeal melanocytic neoplasms associated with neurocutaneous melanosis derive from a single postzygotic somatic mutation in NRAS [53,54] or BRAF [55]. In one case of isolated leptomeningeal melanocytosis, an NRAS mutation was identified [56]. TERT promoter mutations have not been investigated in this context thus far. Progressive diffuse meningeal melanocytic neoplasms show an increase in copy-number variations similar to those described in cutaneous melanoma [57,58]. Alternatively, the amplification of the mutated NRAS gene has been described [59]. CSF molecular testing has been proven feasible to achieve an early diagnosis of primary diffuse leptomeningeal melanomatosis [41]. In three cases, the diagnosis was solely achieved by CSF cytology [27,40,60]. Baumgartner et al. [41] found an NRAS(Q61R) mutation in both tumor tissue from biopsy and CSF liquid biopsy: the mutant allelic frequency was higher in CSF (3%) as compared with the tumor tissue (1%), suggesting that CSF liquid biopsy could recapitulate the molecular aberrations of primary diffuse leptomeningeal melanomatosis.

## 4. Neuroimaging Diagnosis

Imaging is first needed to exclude a melanoma metastasis in the CNS. Magnetic resonance imaging (MRI) can suspect the melanocytic nature of a lesion, as further explained, but cannot diagnose a primary or secondary origin. Therefore, when a melanocytic tumor is suspected, the existence of systemic disease should always be excluded with a thorough clinical examination and whole-body imaging study that includes 18F-fluorodeoxyglucose positron emission tomography-computed tomography (18F-FDG PET-CT), gastrointestinal endoscopy, as well as skin and eye examinations [41]. A spine MRI should also be carried out to inform on spread along the whole neuraxis.

### 4.1. Computed Tomography (CT)

Computed tomography (CT) is commonly used as the first neuroimaging assessment, especially in emergency settings. On CT, a melanocytic neoplasm is often hyperdense, therefore mimicking hemorrhage [56], and has a variable degree of homogenous contrast enhancement. Calcifications are rare but can be seen in melanocytomas [27,40]. Acute hydrocephalus can be a radiological finding at presentation in leptomeningeal subtypes [60].

### 4.2. Magnetic Resonance Imaging (MRI)

**Figure 2:**
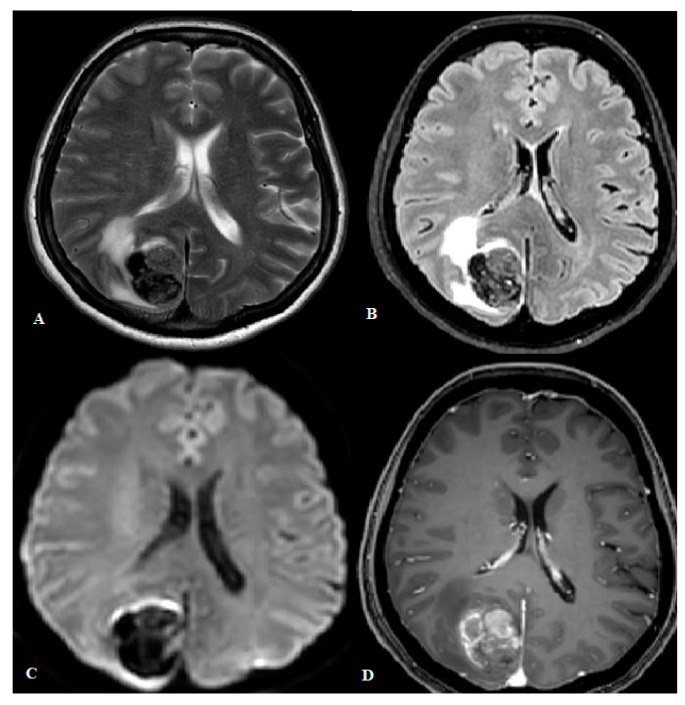
(**A**–**D**) Axial T2w (**A**), FLAIR (**B**), DWI (**C**) and T1w post contrast (**D**) scan of a 67-year-old woman displaying a parietal meningeal melanocytoma.

On MRI, melanin is typically hyperintense on T1 weighted (T1w) sequences (Figure 2) and hypointense on T2 weighted (T2w) sequences, as a reflection of its paramagnetic effects, which shortens T1 and T2 relaxation times [51,61]. Melanocytic lesions also show a substantial signal loss in susceptibility-weighted (SW) sequences [62]. This phenomenon, also known as blooming artefact, seems to originate from deoxyhemoglobin, a strong paramagnetic molecule, rather than melanin pigment content by itself [63], and is probably a result of several micro-bleedings and metal-scavenging melanin properties [63]. Intratumoral microhemorrhages develop over time (median ~3 months) [64] and can be absent in early melanocytic lesions [64]. As a consequence, SW imaging can be negative at initial stages. Of note, the collection of this evidence rises from in vitro and in vivo analyses conducted on melanoma metastases and not on primary melanocytic tumors of the CNS. Nevertheless, it is reasonable to believe that these phenomena also occur in the primary CNS counterpart.

Radiological–pathological correlation studies on melanoma metastases have also shown that the degree of melanocytic content correlates with T1 shortening [63]. This explains why lesions with a poor melanin content (i.e., less than 10%) do not have the typical T1 hyperintensity [65] and are therefore radiologically defined as “amelanotic lesions [63]. Conversely, a clear association between T2w hypo-intensity and melanin content has not been shown [64].

**Figure 3:**
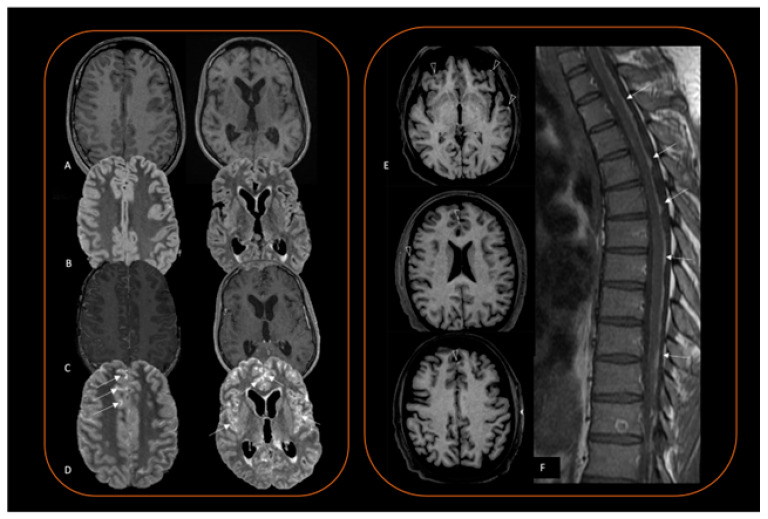
(**A**–**D**) A case of diffuse meningeal melanomatosis in a 14-year-old boy. In the first and second column, (**A**) 3D T1w sequence, (**B**) 3D FLAIR, (**C**) 3D T1w after contrast injection, (**D**) 3D FLAIR after contrast injection. This case displays the ability of delayed 3D FLAIR sequence to show leptomeningeal disease better than T1w post-contrast sequence (some examples of this increased visibility are marked with arrows). (**E**,**F**) Another case of diffuse meningeal melanomatosis (case courtesy of Darío Herrán de la Gala). (**E**) 3D T1w before contrast injection showing small focal areas of spontaneous T1 hyperintensity (arrowheads). (**F**) sagittal T1w sequence showing an extensive medullary involvement of the disease.

MRI also distinguishes between a circumscribed (Figure 2) and a diffuse (Figure 3) lesion pattern but does not inform on the degree of lesion aggressiveness, and does not distinguish a melanoma from a melanocytoma, or a melanocytosis from a melanomatosis. Melanocytoma and melanoma are usually solitary and show a predilection for the posterior fossa, Meckel cave, and cervical spinal cord [18]. These locations seem to reflect the sites in which leptomeninges have a higher concentration of melanocytes [14,66]. In contrast, supratentorial lesions are rarer [67]. In the spinal canal, most of these tumors are in the intradural extramedullary compartment, but occasionally can also be seen extradurally or intramedullary. A retrospective case series of 16 patients with pathologically proven spinal meningeal melanocytomas showed typical melanocytic lesion signal characteristics, influenced by intratumoral hemorrhage, and no peritumoral edema or syringomyelia [20]. Intervertebral foramen can be enlarged, and be erroneously interpreted as a neurogenic tumor [40].

### 4.3. Positron Emission Tomography (PET)

PET-CT shows intense 18F-FDG uptake [68], and informs on CNS disease extension. Moreover, 18F-FDG PET-CT is useful in the case of differential diagnosis with metastatic melanoma for the detection of systemic localizations.

Given the rarity of the disease, many conditions may mimic a primary melanocytic tumor. Extra-axial circumscribed lesions can morphologically resemble a meningioma [69], a sellar location may be indistinguishable from a hemorrhagic pituitary macroadenoma [70,71], while a leptomeningeal spread [36] should evoke more common causes of subacute meningitis (i.e., infective, autoimmune or other tumoral pathology). In the absence of T1 leptomeningeal hyperintensity (Figure 3A), preoperative suspicion is almost impossible. As leptomeningeal proliferation is often present in these patients and results in leptomeningeal enhancement, neuroradiologist should choose to perform a 3D FLAIR delayed post-contrast sequence, which is, to date, the sequence with the highest sensitivity for leptomeningeal contrast enhancement evaluation (Figure 3A–D) [72]. Brain MRI protocol should therefore include 3D T1 spin echo sequence before and after contrast injection, and a 3D FLAIR post-contrast sequence. Susceptibility imaging could also be added to inform on bleeding lesion status. Of note, a spontaneous T1 hyperintensity in a melanocytic lesion may also reflect intratumoral bleeding instead of melanin, as extracellular methemoglobin is strongly paramagnetic, resulting in T1 hyperintensity [73]. However, a hemorrhage has different stages and evolves in time, changing MRI signal characteristics, while melanin signal alone does not change over time [74].

## 5. Treatment Options

### 5.1. Surgery

There is only a handful of papers on the surgical management of primary melanocytic tumors of the CNS, all of them being case reports or small case series. Usually, at the time of surgery, the diagnosis is still unclear. These tumors, when occurring as solitary lesions, are attached to the dura and may be interpreted as meningiomas. Therefore, the surgical strategy is according to meningioma management and comprises decompression of adjacent neural structures and complete removal of the tumor including the involved dura. This simple, meningioma-derived surgical strategy seems to be appropriate for melanocytic tumors of the CNS. In a series of 17 meningeal melanocytic tumors (4 melanocytomas and 13 malignant melanoma) reported by Brat et al. [14], meningeal melanocytomas did not recur after a gross-total resection. Conversely, 8/13 patients with meningeal melanoma recurred after surgery, with 4 being fatal (median survival of 14 months). Of five totally resected meningeal melanomas, four did not recur (median follow up of 26 months). while only one recurred after a median follow up of 26 months. This is also supported by even smaller series comprising five cases, each from Freudenstein et al. [75] and Jaislwal et al. [76], who also recommended a complete resection for either meningeal melanocytomas or melanomas as the main goal of therapy.

As a good practice point, in cases where gross total resection is not indicated due to localization or the extent of the tumor, an open or stereotactic biopsy should be performed to achieve integrated diagnosis.

### 5.2. Radiotherapy

Recommendations for radiotherapy are based on expert opinion according to the available literature and the general principles for the treatment of brain tumors, as the reported cohorts or case-series are too small for data-driven analysis [1,3,77,78]. Decision making should be based on taking the following parameters into account: benign versus malignant histology, circumscribed versus diffuse pattern, complete versus incomplete resection. High-dose radiotherapy is rarely indicated in diffuse melanocytic tumors except as palliative treatment to alleviate symptoms if appropriate. In the largest series by Puyana et al. [79], a total of 54 patients retrieved from the Surveillance, Epidemiology, and End Results database from 1973–2015 were analyzed. Patients received a gross-total resection in 40.7%, a subtotal resection in 7.4%, adjuvant radiotherapy in 46.3%, and chemotherapy in 27.3%. The most favorable outcome was achieved with gross-total or subtotal resection followed by postoperative radiotherapy. No survival benefit was achieved with subtotal resection, radiotherapy or chemotherapy alone. Thus, general agreement exists that radiotherapy should be employed after subtotal resection and should be considered in localized malignant melanoma even after gross-total resection, as a considerable proportion of patients in the Surveillance, Epidemiology, and End Results series had received postoperative radiotherapy [79]. After biopsy only or relapse after gross-total resection, high-dose radiotherapy can be employed to delay complications from further growth, although no impact on overall survival can be expected. Conventional fractionated radiotherapy with up to 60 Gy has been mostly employed, although radiosurgery has been used for selected patients with smaller tumors and up to three lesions [1,3]. Thus, in the postoperative and primary setting 60 Gy in 1.8–2.0 Gy are recommended with a clinical target volume margin of 5–10 mm as in primary malignant tumors of the brain, respecting natural anatomical barriers.

### 5.3. Medical Treatment

Systemic therapies should be considered in the case of residual/progressive disease of meningeal melanocytoma/melanoma, when surgery and/or radiotherapy have been exhausted, or in patients with primary diffuse leptomeningeal melanocytosis or melanomatosis. Considering the diffuse infiltrative pattern of primary meningeal melanocytic tumors and the significant radio-resistance, medical treatments are considered as salvage therapies. Nevertheless, the role of systemic therapies, including traditional chemotherapy, targeted therapy, or immunotherapy, remains to be clearly defined. Of note, uveal melanomas share different activating mutations with melanocytic tumors, including GNAQ/GNA11, BAP1, or SF3B1, with the emergence of targeted and epigenetic therapeutic strategies for the metastatic setting [80]; however, clinical trials or prospective studies focused on melanocytic tumors are lacking and their management is still based on case-reports or applying lines of treatment used in leptomeningeal diffusion from extracranial metastatic melanoma.

Systemic therapy has displayed a limited efficacy in terms of disease control [25,26,28,29,30,31,32,33,34,35,36,37,38,39,41,81,82], regardless of the type of treatment, including either intrathecal chemotherapy via Ommaya reservoir with etoposide, cytarabine, and topotecan, and systemic therapies, such as interferon alpha, temozolomide, everolimus, trametinib, nivolumab alone or in association with ipilimumab (Table 1).

Anectodical reports of efficacy and prolonged overall survival have been reported with different chemotherapy regimens, including temozolomide plus cisplatin plus vindesine plus PEG-interferon-alfa-2B (11 months) [32], temozolomide in association with carboplatin and etoposide (24 months) [34], cisplatin in combination with dacarbazine and whole-brain radiotherapy (12 months) [36], and temozolomide as first line treatment followed by intravenous fotemustine at recurrence (24 months) [38] (Table 1).

Whether the activity of the main treatments used in metastatic melanoma, such as check-point inhibitors with Programmed Cell Death 1 (PD-1) and/or Cytotoxic T-Lymphocyte Associated Protein 4 (CTLA-4) blocking antibodies, or the combination of B-Raf Proto-Oncogene (BRAF)- and Mitogen-Activated Protein Kinase Kinase 1 (MEK)-inhibitors, may be an effective therapeutic option for PMMT remains to be elucidated. Overall, the benefit from immunotherapy or BRAF/MEK inhibitors is weak according to a few case reports on primary meningeal melanocytic tumors [33,38,39,40,41].

In conclusion, a definitive advantage of one specific therapeutic approach over another could not be concluded, and despite the treatment efforts, the median overall survival remains poor, ranging from 4 to 12 months from the diagnosis.

## 6. Discussion

Primary meningeal melanocytic tumors are the paradigm of ultra-rare brain tumors and represent a challenge in terms of diagnosis and treatment. These entities must be differentiated from metastatic melanoma and some clinical factors may suggest the primary CNS origin of the disease; in fact, metastatic melanomas typically develop in older patients (>50 years) with a previous history of cutaneous melanoma and are often found in the context of diffusely metastatic disease conferring a poorer prognosis. Furthermore, molecular analysis is helpful for the diagnosis of primary meningeal melanocytic tumors. The detection of GNAQ, GNA11, PLCB4, and CYSLTR2, as well as the methylation profiling, can support the diagnosis of these tumors as primary CNS neoplasms, and discriminate them from other melanocytic lesions, including brain or leptomeningeal metastases from melanoma, where nearly 50% of cases harbor HRAS, KRAS, BRAF, or KIT mutations. Primary meningeal melanocytic tumors must be differentiated from malignant melanocytic nerve sheath tumors, which typically arise from paraspinal or visceral autonomic nerves and consist of fascicular to sheet-like proliferation of variably pigmented, relatively uniform plump spindled cells with co-expression of S100/SOX10 and melanocytic markers or PRKAR1A mutation [83]. Furthermore, some additional molecular alterations, such as SF3B1, EIF1AX, and BAP1 mutations, confer a significant aggressive course in meningeal melanoma, while both primary diffuse leptomeningeal melanocytosis or melanomatosis are often NRAS mutated and rarely BRAF mutated.

Neuroimaging suggests a melanocytic content, displays the extension and patterns of progression, but does not inform clinicians regarding the aggressiveness of the disease. Of note, primary meningeal melanocytic tumors may be difficult to discriminate from meningiomas, as they are both dural-based. Overall, the rarity of this tumor type and the impossibility of distinguishing between primary and secondary melanoma preclude the preoperative radiological diagnosis. Intratumoral hemorrhage largely influences the imaging appearance of a melanocytic lesion and can mislead CT and MRI interpretation. Nevertheless, imaging is requested to exclude a systemic disease and can narrow the differential diagnosis by suggesting a melanocytic lesion. In addition, it shows a circumscribed or diffuse lesion pattern, thus driving surgical decisions and pathological integrated diagnosis.

Despite the advances in molecular profiling of primary meningeal melanocytic tumors, strong evidence regarding optimal management is lacking and based on anectodal reports or small cohorts. Overall, the prognosis is better for circumscribed meningeal melanocytoma and melanoma, especially in the case of gross-total resection. Conversely, the prognosis of diffuse meningeal melanocytic tumors is remarkably poor. When a residual or progressive primary melanocytic tumor is found, radiotherapy should be considered. The employment of radiotherapy is still a topic of debate as the recurrence rates in patients who have undergone complete and incomplete resections can be up to 71% within 5 years and 50% within 1 year, respectively [9,18,84]. Some authors suggest adjuvant radiotherapy in cases of both gross-total or subtotal resection of primary meningeal melanocytic tumors, especially in solitary and localized lesion [9,84]. Alternatively, a careful surveillance should be performed, and in case of recurrence, whenever possible, re-operation should be considered. If not feasible, radiotherapy is advised [85]. Systemic therapies have limited impact and are delivered based on the activity of the metastatic melanoma with modest benefit. Although primary diffuse melanocytic tumors harbor distinct molecular alterations, there are no available targeted therapies with a significant impact, and the choice of systemic treatment is based on local protocol or the expertise of each physician.

Considering the rarity and the complexity of these entities, a multidisciplinary approach, incorporating clinical, radiological, and histopathological expertise for accurate diagnosis is strongly encouraged. Moreover, these patients should be referred to centers with recognized expertise in the management of ultra-rare entities for tailoring treatments and improve the outcome.

## 7. Conclusions

An improvement in the knowledge regarding the correlations between histology, molecular features and clinical course should be an aim in order to achieve a precise and early diagnosis. This issue could be addressed through strong collaboration and a multidisciplinary network to build and maintain dedicated platforms and registries for sharing patients’ characteristics, therapeutic interventions, outcomes, and correlations with the molecular profile of primary meningeal melanocytic tumors.

## Figures and Tables

**Table 1:** Type of treatments in cases of residual/progressive primary diffuse leptomeningeal melanocytosis or melanomatosis.

Authors	Sex/Age (Years)	Symptoms	Distinct Mass on Imaging Workup	Diagnosis	Medical Treatment	OS
Nicolaides et al., 1995 [25]	M/5	Increased ICP; headache, nausea, vomiting, intermittent fever, anorexia and weight loss	Yes, left parietal lobe	Parietal biopsy	Local PNET protocol	Not reported
Makin et al., 1999 [26]	M/5	Increased ICP	No, only leptomeningeal spread	Meningeal biopsy	Carboplatin + etoposide + vincristine	6 months
Tosaka et al., 2001 [27]	M/20	Increased ICP, hydrocephalus	No, only leptomeningeal spread	Second CSF tap test (cytology)	Dacarbazine + numistine + vincristine + intrathecal methotrexate	4.5 months
Bocquillon et al., 2010 [28]	F/32	Increased ICP, transient hemiparesis	No, only leptomeningeal spread	Meningeal biopsy	Fotemustine	4 months
Michael et al., 2010 [29]	M/75	Vomiting, poor balance, gait disturbance, cognitive decline	Yes, left parietal lesion	Gross-total section	Goserelin+ bicalutamide + docetaxel	16 days
Lee et al., 2013 [30]	M/17	Headache, nausea, vomiting	Yes, left heel	Left heel biopsy	WBRT + dacarbazine + carmustine + cisplatin + tamoxifen	Not reported
Trinh et al., 2014 [31]	M/51	Headache, nausea, vomiting, hip pain, fatigue	Yes, multinodular diffuse parenchymal enhancement	Meningeal biopsy	CSI + temozolomide	3 months
Angelino et al., 2016 [32]	F/2	Mild, intermittent diplopia	No, only leptomeningeal enhancement	Meningeal biopsy	Temozolomide + cisplatin + vindesine + PET-interferon alfa-2B	11 months
Carey et al., 2016 [33]	M/36	Headache, bilateral leg numbness, bilateral decreased visual acuity	No, only leptomeningeal enhancement	Meningeal biopsy	Ipilimumab	1 month
Szathmani et al., 2016 [34]	F/5	Headache, vomiting, seizures	Yes, bulky left parietal lesion	Left parietal biopsy	Etoposide + temozolomide + carboplatin	24 months
Jacob et al., 2016 [35]	M/55	Headache, nausea, fatigue	No, only leptomeningeal spread	Meningeal biopsy	Temozolomide	Not reported
Aslan et al., 2018 [36]	M/21	Headache, vomiting, fever	No, only leptomeningeal enhancement	Meningeal biopsy	Cisplatin + dacarbazine + WBRT	12 months
Aslan et al., 2018 [36]	F/44	Nausea, vomiting, weight loss, mental slowness, poor concentration, impairment in short-term memory	No, only leptomeningeal diffusion	Meningeal biopsy	Temozolomide	4 months
Fujimory et al., 2018 [37]	M/37	Headache	Yes, left temporal lesion	Partial resection	1st: WBRT + vemurafenib 2nd: nivolumab	5 months
Nicolotto et al., 2018 [38]	M/36	Back pain, headache, mental confusion, cervicalgia, tinnitus, facial paresis	Yes, lumbar (L2) bulky lesion with spinal compression	Gross-total resection	1st: temozolomide 2nd: fotemustine	24 months
Eichberg et al., 2019 [39]	F/44	Mental confusion, left facial palsy, slurred speech, left hemiplegia, hydrocephalus	No, only leptomeningeal diffusion	Meningeal biopsy	Nivolumab + ipilimumab	4 months
Tamura et al., 2020 [40]	F/49	Hydrocephalus	No, only leptomeningeal diffusion	3rd CSF cytology	Nivolumab + ipilimumab	4 months
Baumgartner et al., 2020 [41]	M/15	Increased ICP, bilateral papilledema	Yes, parieto-occipital lesion	Parenchymal biopsy	1st: trametinib 2nd: tametinib + everolimus 3rd: nivolumab + ipilimumab 4th: WBRT	6.5 months

M: male; F: female; ICP: intracranial pressure; PNET: primary neuroectodermal tumor; CSF: cerebrospinal fluid; WBRT: whole-brain radiotherapy; CSI craniospinal irradiation.

## Data Availability

No new data were created or analyzed in this study. Data sharing is not applicable to this review.

## Abbreviations

BAP1	BRCA1-associated protein-1
BRAF	V-raf murine sarcoma viral oncogene homolog B
CNS	Central nervous system
CSF	Cerebrospinal fluid
CTLA4	Cytotoxic T-lymphocyte associated protein 4
CYSLTR2	Cysteinyl leukotriene receptor 2
EIF1AX	Eukaryotic translation initiation factor 1A X-linked
EURACAN	European Network for Rare Cancers
FLAIR	Fluid-attenuated inversion recovery
GNAQ	G protein subunit alpha q
GNA11	G protein subunit alpha 11
Gy	Grey
HPF	High-power field
HRAS	Harvey rat sarcoma virus
KIT	KIT proto-oncogene
KRAS	Kirsten rat sarcoma virus
MEK	Mitogen-activated protein kinase 1
MRI	Magnetic resonance imaging
PD-1	Programmed cell death 1
PLCB4	Phospholipase C Beta 4
SF3B1	Splicing factor 3b subunit 1
TERT	Telomerase reverse transcriptase
WHO	World Health Organization
18F-FDG PET-CT	18F-fluorodeoxyglucose positron emission tomography-computed tomography
PRKAR1A	Protein kinase cAMP-dependent type I regulatory subunit alpha
S100	Calcium binding protein
SOX10	SRY-box transcription factor 10
CT	Computed tomograph
